# How much genetic information in RNA form can be protected by a CCMV virus-like particle?

**DOI:** 10.1371/journal.pone.0336376

**Published:** 2025-12-10

**Authors:** Ana Luisa Duran Meza, Sophie-Christine Porak, Abigail Chapman, Charles M. Knobler, William M. Gelbart

**Affiliations:** 1 Department of Chemistry and Biochemistry, University of California, Los Angeles, California, United States of America; 2 Molecular Biology Institute, University of California, Los Angeles, California, United States of America; 3 California Nanosystems Institute, University of California, Los Angeles, California, United States of America; East China Normal University School of Life Sciences, CHINA

## Abstract

The capsid protein of cowpea chlorotic mottle virus (CCMV) has been used extensively for in vitro packaging of heterologous RNA. The associated virus-like particles (VLPs) are spherical with a 3nm-thick/28nm-diameter protein shell and are therefore limited in the amount of RNA they can package. As shown in earlier work, when RNA lengths are longer than ~3500nt the RNA is no longer self-assembled exclusively into a single VLP. Rather, it is shared by two or more 28nm-diameter capsids in the form of doublets, triplets, and higher-order multiplets, with the RNA threaded through the naturally- occurring ~1.5nm-diameter holes in the 180-subunit/icosahedrally-symmetric protein shells. Consistent with this fact we find in the present work that 3500nt is the maximum length of packaged RNA that is fully protected under strong RNase digestion conditions.

## Introduction

We are concerned here with in vitro reconstituted non-infectious virus-like particles (VLPs) that are self-assembled nucleocapsids formed from purified viral capsid protein and heterologous – non-viral – RNA. Essentially all VLP syntheses of this kind to date have been carried out with capsid protein from the plant viruses cowpea chlorotic mottle virus (CCMV) [1–5], brome mosaic virus (BMV) [6–9], and tobacco mosaic virus (TMV) [10–12], and from the bacterial virus MS2 [13–15]. With mRNA vaccines and therapeutics currently attracting widespread interest [16–18], VLPs are promising candidates to pursue as alternatives to lipid nanoparticles [19–21] for targeted mRNA delivery because of their stoichiometric preciseness, enhanced thermodynamic stability, and ease of functionalization.

In this work, we focus on VLPs derived from CCMV capsid protein (CP) self-assembled around RNA molecules of different lengths, to determine the length limit of effective encapsidation. It is known that the capsid protein of CCMV is capable of encapsidating a 100-fold range of RNA lengths, but when the RNA is longer than its largest viral-gene length, ~3200 nt, the RNA gets packaged into multiplets, i.e., two or more capsids surround a single RNA molecule. More explicitly, Cadena-Nava et al. [1] demonstrated that CCMV CP can efficiently package RNA molecules ranging in length from 140 to 12000 nt as long as the protein:RNA mass ratio is greater than the stoichiometric value (~4) for 180 CPs. Further, consistent with the 28 nm-diameter preferred by the CP, a pair of capsids – a ‘‘doublet’’ – is found to share a single RNA molecule when its length is ~6000nt; similarly, triplets and quadruplets of capsids share in the packaging of single ~9000nt and ~12000nt RNA molecules, respectively. Starting with single capsids that form for ~3000nt-packaged-long RNAs, the fraction of singlets decreases upon increase in the length of RNA being packaged and the self-assembled structures involve predominantly a mix of singlets and doublets, then doublets and triplets, etc., as one passes through the succesive threshholds of ~6000nt and ~9000nt. Note that the CCMV genome is multipartite, consisting of: RNA1 (~3200 nt) and RNA2 (~2800 nt), each of which is packaged into separate but identical capsids, and RNA 3 (~2100 knt) and RNA4 (~800 knt) that are co-packaged in a 3rd identical capsid [22,23]. These facts reflect the strong preference of the capsid protein for encapsidating a particular amount (~3000 nt) of RNA in ~28-nm-diameter, and account for the multiplets of identical wildtype capsids that form when longer molecules are self-assembled with capsid protein. The RNA shared by two or more capsids is threaded through the naturally occurring interstices between capsomers or the central holes of hexamers. The multiplets arise from two or more independent capsid-forming nucleation events taking place at different points on an ‘overlong’ RNA molecule, with the corresponding capsids ‘closing’ next to one another at a single-stranded portion of the RNA, i.e., at a sequence that isn’t involved in duplex formation. Accordingly, the naturally occurring ~1.5 nm-diameter ‘holes’ at the interstices between pentamers and hexamers [24] – holes that are too small to accommodate double-stranded RNA – become the “linking” portion connecting two capsids in a multiplet.

Here we show that 3500 nt is the limiting length of RNA molecules that can be efficiently assembled into CCMV VLP singlets and become fully protected against concentrations of RNase A significantly greater than that sufficient to digest “naked” RNA. We also show that CCMV VLP multiplets are partially RNase-resistant up to RNase:RNA mass ratios well above typical physiological values [25]. This result suggests that therapeutic RNAs longer than 3500 nts, while not packageable exclusively into CCMV VLP singlets, can nevertheless be used in multiplet VLP form for in vivo applications. For RNA lengths up to 3500 nt – specifically, for the 3175 nt, 3234 nt, and 3500 nt molecules synthesized here – spontaneous assembly into single capsids is associated with the fact that these molecules have essentially the same size as the wildtype virion [26], so that little change in their secondary/tertiary structure is expected upon their packaging.

The susceptibility of multiplet VLPs to nuclease digestion arises because there is a portion of RNA threaded between capsids for all RNA molecules that are too long to be accommodated by a single capsid. The extent to which this happens has been roughly quantified in experiments by Duran-Meza, et al. [6] They treated assembly mixes with RNase A at concentrations greater than that in blood serum, extracted the RNA from the capsids, and determined the fraction of RNA that had not been digested. From their studies, which were carried out with seven RNAs ranging in length from 3234 to 11,703 nt – in particular, lengths of 3234, 4196, 4413, 4638, 6395, 8935, and 11,703nt – it was found that only the packaged 3234nt-long RNA was completely protected against nuclease, from which we deduce that the limiting length of protected RNA is somewhere between 3234 and 4196nt. In the present work we determine this length more precisely by examining several molecules in the small range of lengths between 3175 and 4026 nt at a resolution of 200–300nts, allowing us to establish the packaging limit to be 3500 ± 100 nt. Further, for longer molecules, where a significant fraction is packaged into doublets, we find that most of the packaged RNA – in contrast with “naked” RNA – survives intact when incubated at physiological RNase A concentrations.

## Materials and methods

### RNA transcription and purification

Restriction enzymes from New England Biolabs (Ipswich, MA) were used as recommended by the manufacturer. The plasmids (sequences provided on request) were grown and purified using QIAprep (Qiagen DEU) following the manufacturer’s specifications. A Nodamura-RNA1-plus-ferritin-gene plasmid was linearized with NheI, SbfI, BsteII, AgeI and XbaI restriction enzymes to produce RNA transcripts with lengths of 3175, 3500, 3697, 3799 and 4026 nt, respectively. A Nodamura-RNA1-plus-EYFP- gene plasmid was linearized with AgeI and XbaI to produce RNA transcripts 3970 and 4197 nt in length, and a Nodamura-RNA1-plus-EYFPgene-TMV-OAS-sequence plasmid was linearized with XbaI to produce an RNA transcript of 4433 nt. Finally, a plasmid containing a full-length cDNA form of BMV RNA1 was treated with BamHI to produce an RNA transcript of 3234 nt. All of the templates were purified using a QIAquick PCR Purification Kit (Qiagen DEU) following the manufacturer’s specifications, linearized, and used as templates for *in vitro* transcription using a MEGAscript T7 Transcription Kit (Thermo Fisher Scientific, Waltham, MA).

The RNA was purified using a QIAGEN RNeasy Mini Kit (Germantown, MD), except for the 3500 nt RNA transcript of the Noda-RNA1 ferritin plasmid linearized with SbfI, which showed two bands and was further purified by electroelution. The 3500 nt RNA was loaded onto a 0.8% agarose gel and run at 100 V for 1.5 h. The portion of the gel containing the desired length was excised and placed in a dialysis bag with TAE buffer. A voltage of 100 V was applied for 30 min, which caused the RNA to migrate out of the gel into the dialysis bag buffer. The RNA was purified with a 100 kDa Amicon centrifuge filter (0.5 mL) at 4000 g for 4 min for each flow. The RNA was washed with milliQ water (3 x the original sample volume) at 4000 g for 4 min. It was eluted at 1000 g for 1 min and characterized by UV-Vis spectroscopy with a NanoDrop spectrophotometer.

### Capsid protein expression and purification

CCMV CP was expressed and purified from *E. coli* as described by Karan, et al. [11] Competent cells of *E. coli* strain Rosetta 2 BL21 (New England Biolabs, Ipswich, MA) were transformed with the T7-7 × HisTag-CCMVCP construct for CCMV capsid protein expression (sequence provided upon request). The transformed cells were grown in LB medium (Thermo Fisher Scientific, Waltham, MA) supplemented with ampicillin and chloramphenicol (Goldbio, St Louis, MO), at 37°C with constant agitation until an optical density of 0.6 absorbance units was attained. Recombinant expression of the CCMV CP was induced after the addition of isopropyl-d-1-thiogalactopyranoside (IPTG) (Goldbio, St Louis MO), at a final concentration of 1 mM and incubated for 16 h at 20°C with constant stirring. Induced cells were collected by centrifugation at 15,000 g for 10 min at 4°C, with the pellet stored at −80°C until purification. Cell pellets were resuspended in salt lysis buffer (1 M NaCl, 50 mM Tris-HCl, 5 mM TCEP) (Goldbio, St Louis MO). The resuspended cells were lysed by sonication and homogenized in a Emulsiflex homogenizer (Avestin Emulsiflex C-3). The cell lysate was centrifuged at 12,000 g for 45 min at 9°C and the supernatant was collected. The protein was purified using the 7x histidine tag, by running the cell lysate over a nickel resin column (Thermo Scientific, Waltham, MA) eluted with 50mM imidazole (Thermo Scientific, Waltham, MA) and stored at 4°C. The histidine tag was cleaved using pro- TEV protease. Finally, the purified protein was dialyzed using a 6–8 kDa dialysis membrane (SpectraPor S/P 1 Dialysis Membrane; Thermo Fisher Scientific, Waltham, MA) into buffer B (1 M NaCl, 20 mM Tris pH 7.2, 1 mM EDTA, 1 mM DTT, 1 mM PMSF) (Sigma Millipore, U.S.A) and aliquoted at 1 µg/mL. For further use the protein was flash frozen in liquid nitrogen and stored at −80°C until ready to use, at which point it was defrosted on ice and stored at 4°C for up to two weeks. The purity of the CP was determined by UV/Vis spectroscopy (Nanodrop 2000, Thermo Fisher Scientific, Waltham, MA). The concentration of the CCMV CP was determined at A280 using an extinction coefficient of ε = 1.27 μL/μg cm, and only proteins with a A260/A280 ratio < 0.62 were used for assembly.

### *In vitro* assembly of VLP

For assembly of CCMV VLPs, RNAs were mixed with the CP at a protein/RNA mass ratio of 1:4.2 in protein storage buffer (1 M NaCl, 20 mM Tris pH 7.2, 1 mM EDTA, 1 mM DTT, 1mM PMSF) and then dialyzed overnight for 12 h against RNA assembly buffer (50 mM Tris pH 7.2, 50 mM NaCl, 10 mM KCl, 5 mM MgCl_2_, 1 mM DTT) at 4°C [3].

### RNA extraction from VLPs

RNA was extracted from VLPs with a QIAamp Viral Mini Kit (Qiagen DEU) following the manufacturer’s specifications, and eluted in MilliQ water. The concentration was determined by UV/Vis Spectroscopy.

### RNase A treatment

The assembled VLPs were treated with Rnase A (Thermo Scientific) for 30 min at 37°C at different mass ratios of RNA:RNase A. Digestion of RNA was stopped by the addition of RNase inhibitor (Thermo Scientific) and the sample was washed through a 100 kDa MW-cutoff Amicon filter to purify the remaining RNase-resistant VLPs. The purified VLPs were characterized by UV-Vis spectroscopy.

### Agarose gel

The purity and integrity of the RNA were evaluated using electrophoresis in agarose gels stained with GelRed (Biotinum). 300 ng of each sample were loaded on a 1.2% agarose gel and subjected to electrophoresis for 2 h 15 min at 100 V. The transcripts were compared with the Millenium RNA Markers ssRNA ladder (Thermo Fisher).

### TEM analysis of VLPs

6-μL aliquots of purified VLPs (0.025 μg/μL) were spread onto glow-discharged copper grids (400-mesh) coated with parlodion and carbon. After 1 min, the grids were blotted with Whatman filter paper, and then stained with 2% (w/v) uranyl acetate (6 μL) for 1 min. Micrographs were acquired using a Tecnai G2 TF20 High-Resolution electron microscope (FEI, USA) with an accelerating voltage of 200 kV. Images were collected at 3–4 μm underfocus with a TIETZ F415MP 16-megapixel CCD camera (4000 by 4000 pixels, pixel size 15 μm).

## Results and discussion

### Incrementally larger RNAs ranging from 3175nt to 4433nt can be in vitro transcribed in comparable numbers

**Fig 1:**
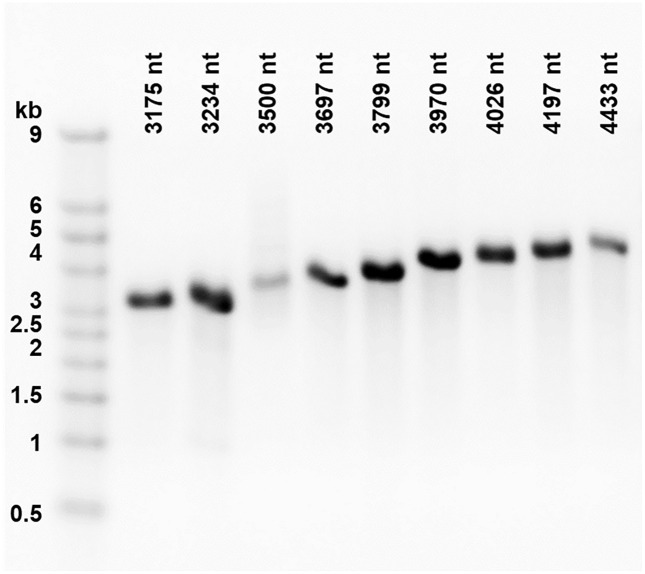
RNA transcription. The purity and integrity of the RNA transcripts were evaluated by electrophoresis with agarose gels stained with GelRed^TM^. 300 ng of each sample were loaded on a 1.2 % agarose gel and subjected to electrophoresis for 2 h 15 min at 100 V. The gel is arranged by increasing length of the RNA transcripts, and the transcripts are compared with the Millenium^TM^ RNA Markers ssRNA ladder (lane 1).

The purified in vitro transcribed RNAs were run in an agarose gel, as shown in Fig 1 in which lane 1 is an RNA ladder and the other lanes are RNA molecules of length 3175, 3234, 3500, 3697, 3799, 3970, 4026, 4197 and 4433 nt, with bands appearing at the corresponding positions.

### RNA can be extracted from CCMV VLPs without degradation

**Fig 2:**
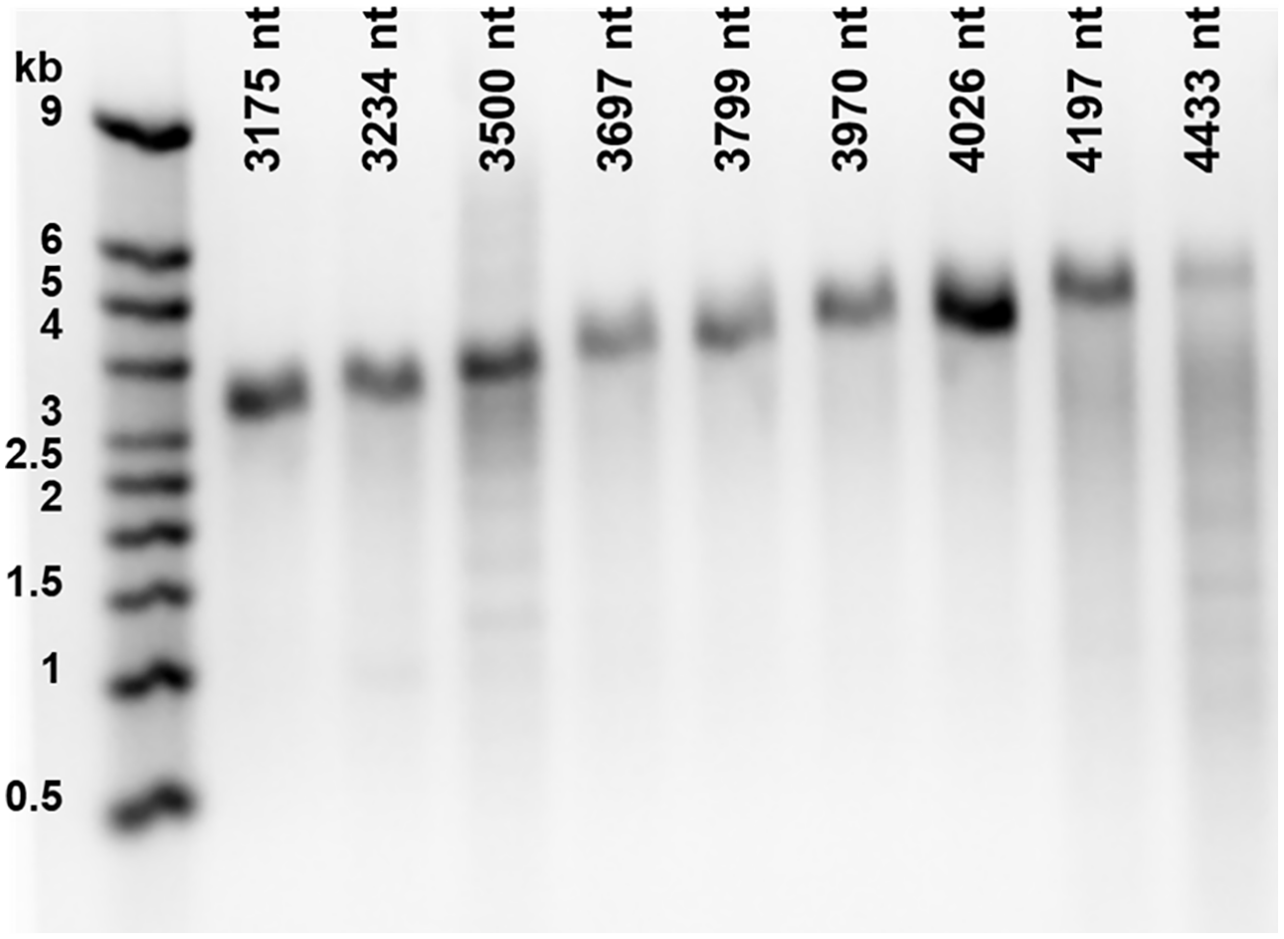
RNA extracted from CCMV VLPs. RNA transcripts were visually evaluated using electrophoresis on agarose gels stained with GelRed. RNA was extracted from CCMV VLPs using a Qiagen viral RNA mini kit. 300 ng of each sample were loaded on a 1.2% agarose gel and subjected to electrophoresis for 2 h 15 min at 100 V. The RNA is compared with the Millenium RNA Markers ssRNA ladder (lane 1). These results show that, apart from some degradation due to extraction handling, all molecules run to their appropriate full-length band positions (as in Fig 1).

Each of nine RNAs with lengths ranging from 3175 to 4433 nt were assembled into CCMV VLPs, which were then purified, concentrated and characterized by UV-Vis spectroscopy. To recover the RNA packaged by capsid protein it was extracted from the VLPs using a Qiagen viral RNA mini kit and characterized by agarose-gel electrophoresis. The results in Fig 2 demonstrate that the RNA was recovered without degradation, as the molecules run to their appropriate band positions and show the same migration pattern as in Fig 1.

### RNA extracted from *RNase A-treated* CCMV VLPs is intact only up to a certain length of packaged RNA

**Fig 3:**
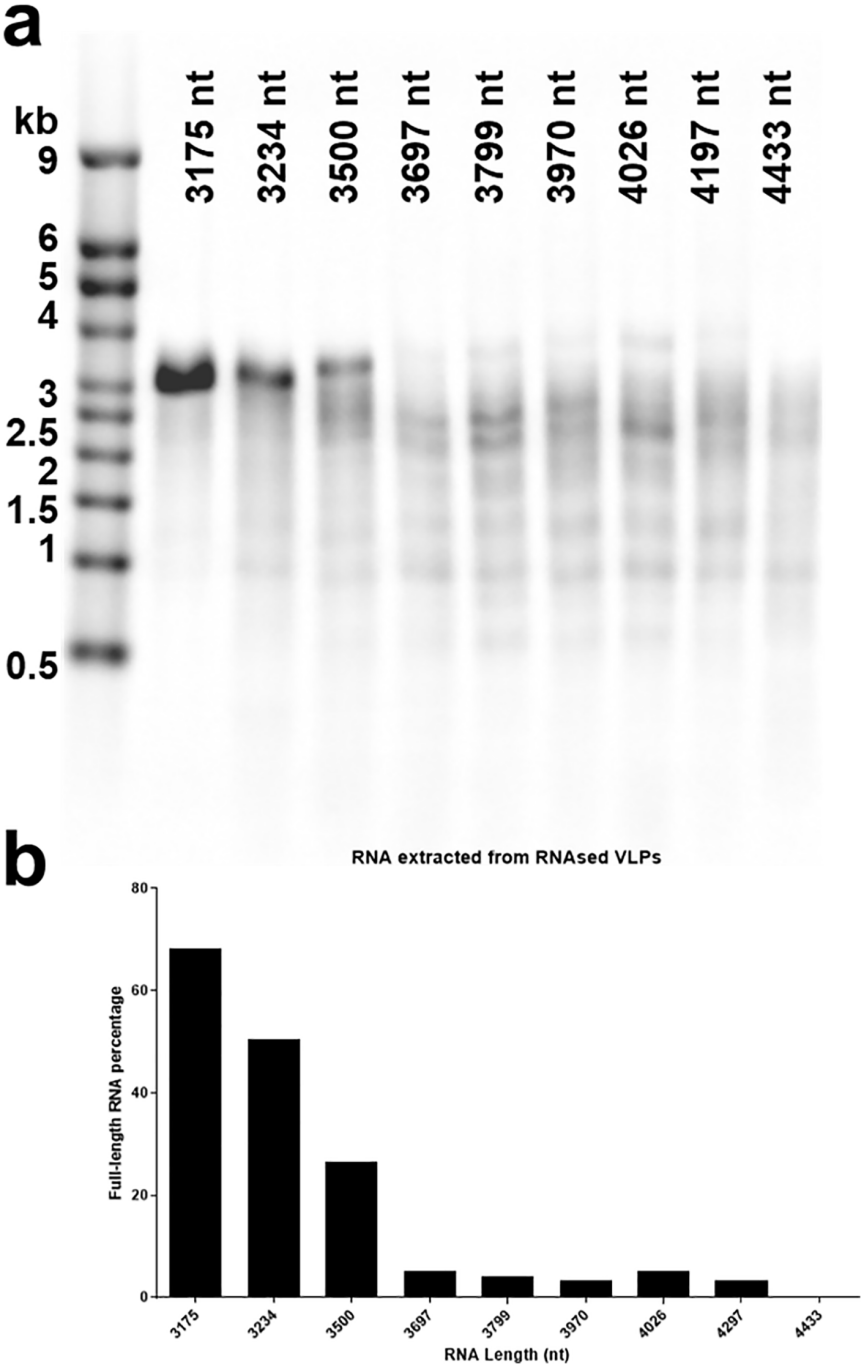
RNA extracted from RNase A-treated CCMV VLPs. a. RNA was extracted from RNase A-treated CCMV VLPs using a Qiagen viral RNA mini kit. 500 ng of each sample were loaded on a 1.2% agarose gel and subjected to electrophoresis for 2 h 15 min at 100 V. The RNA band positions were compared with the Millenium^TM^ RNA Markers ssRNA ladder (lane 1). Lanes 2–4 show strong bands at the expected length. Lanes 5–10 show degraded RNA, indicating that the RNA was not fully encapsidated by CP and thus not protected from RNase A. **b.** Percentage of RNA extracted from RNase-treated capsids that runs at full length, determined from densitometry traces of the lanes in gel 3a (see text).

CCMV VLPs containing the synthetic RNAs were treated for 1 h with 0.11 μg of RNase A for every μg of RNA. To determine how the encapsidated RNA was affected by RNase A treatment, electrophoresis of the RNA extracted from the VLPs was performed on agarose gels, see Fig 3a. The results indicate that packaged RNAs with lengths up to 3500 nt are significantly protected against RNase at this concentration (0.11 mg RNase A:1 mg RNA). A common degradation pattern (distinct bands formed between ~3 and 0.5 kb) is observed, likely due to the shared sequence of the cDNA backbones that were used for in vitro RNA transcription. More explicitly, all of the cDNA backbones contain the sequence for Nodamura virus RNA 1, which has a size of 3204 nt and is the first sequence to be transcribed after the T7 promoter. The corresponding homology over the large part of the total RNA length implies shared secondary structure formation and thus similar degradation patterns upon RNase A treatment.

Quantification of the extent of survival of full-length RNA against a 0.11:1 RNase:RNA treatment is shown as a percentage in the bar graph of Fig 3b, as determined from densitometry traces of lanes 2–10 in Fig 3a. After using ImageJ to make a background correction, this percentage is calculated for each lane as the fraction of total intensity associated with the band corresponding to the position of the full-length RNA for that lane. A drop from double-digit to single-digit percentage is seen to occur for molecules longer than 3500nt.

### TEM images show multiplets are turned into singlets by RNase treatment

**Fig 4:**
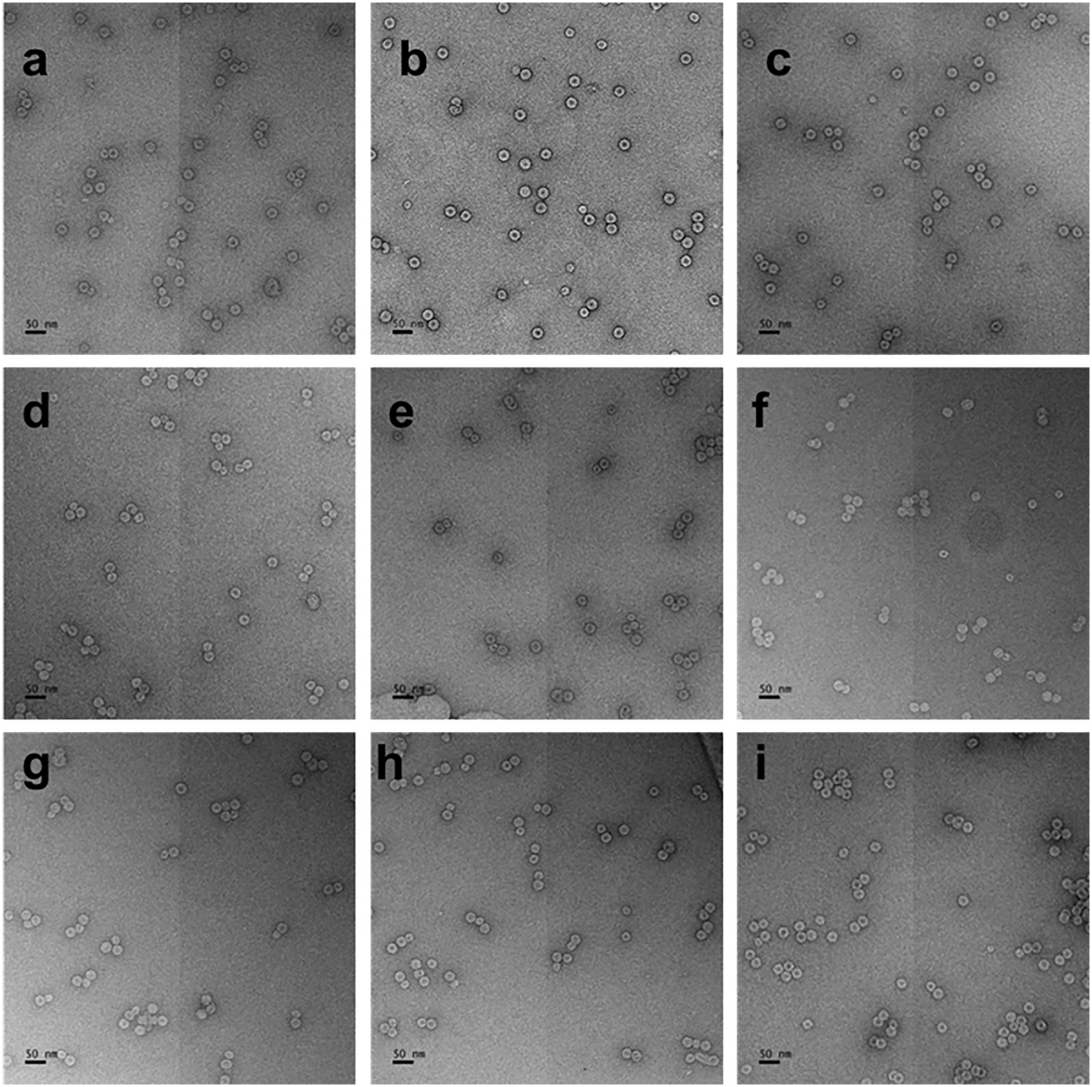
TEM images of VLPs before RNase A treatment, stained with 2% uranyl acetate. a. 3175, b. 3234, c. 3500, d. 3697, e. 3799, f. 3970, g. 4026, h. 4197, i. 4433nt. Scale bars indicate 50nm.

**Fig 5:**
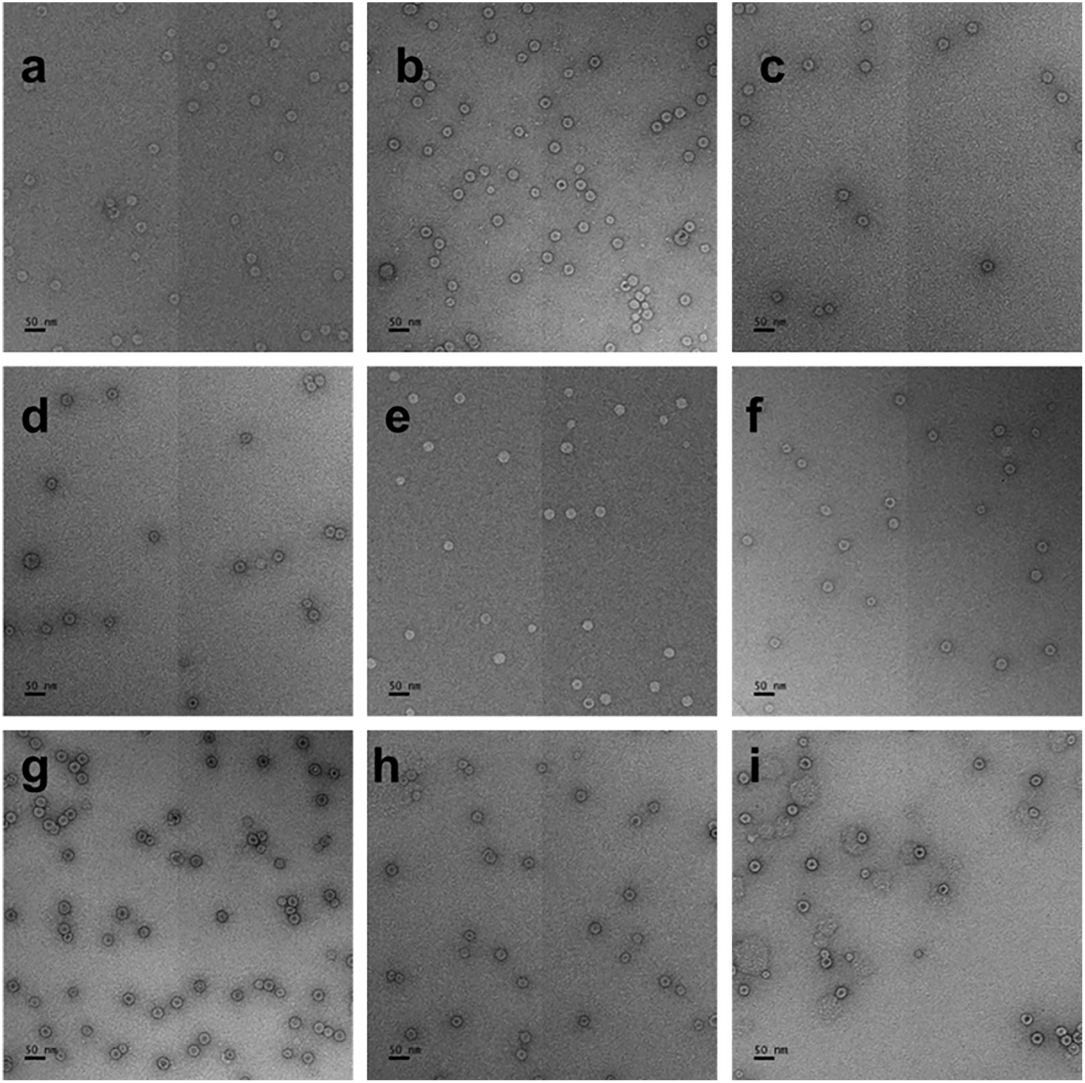
TEM images of VLPs after RNase A treatment, stained with 2% uranyl acetate. a. 3175, b. 3234, c. 3500, d. 3697, e. 3799, f. 3970, g. 4026, h. 4197, i. 4433 nt. Scale bars indicate 50nm.

The effect of RNase on the VLPs can be directly observed in the TEMs shown in Figs 4 and 5. Fig 4, in accord with the previous studies [1], displays the untreated assemblies, showing a progression from singlets to multiplets with increasing RNA length. The multiplets can be distinguished from overlapping singlets by dilution studies, which show that for these lengths – 3175–4433nt – the assemblies consist largely of singlets and doublets, with doublets constituting at most about 30% for the longest RNAs. These doublets “survive” strong dilution “treatment”, whereas after RNase treatment only singlets appear.

### VLP-RNase titrations establish RNA protection limit in multiplets

**Fig 6:**
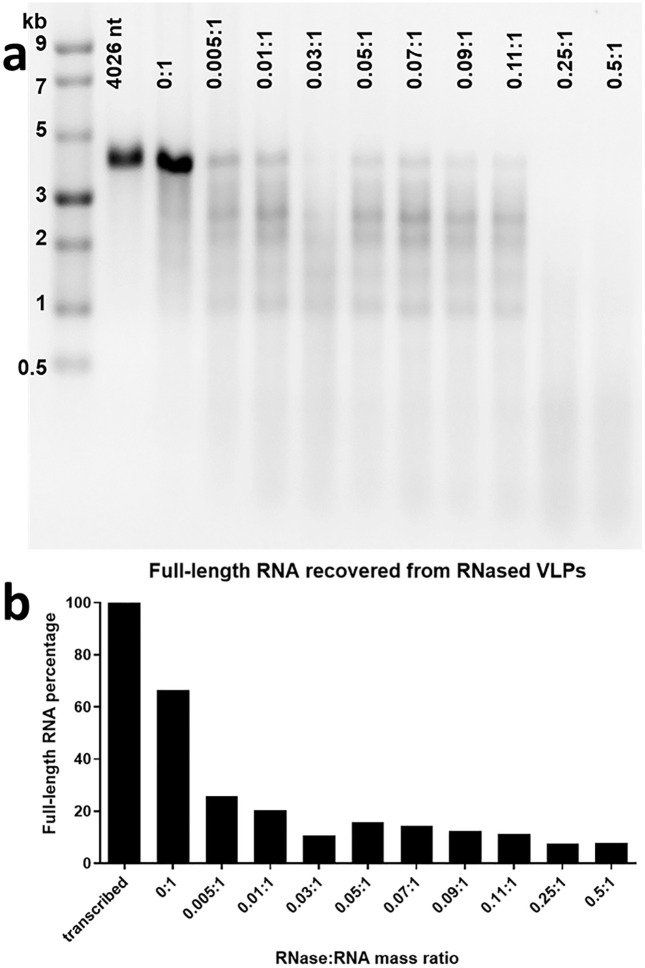
RNA extracted from CCMV VLPs that have been packaged with 4026nt- long RNA, and RNase-treated at different RNase:RNA mass ratios. a. RNA was extracted from RNase A-treated CCMV VLPs using a Qiagen viral RNA mini kit. 500 ng of each sample were loaded on a 1.2% agarose gel and subjected to electrophoresis for 2 h 15 min at 100 V. The RNA was compared with the NEB^TM^ RNA Markers ssRNA ladder (lane 1). Lane 2 is the in vitro transcribed RNA. Lanes 3-12 show the RNA extracted after treatment with 10 different mass ratios of RNAse A. **b.** Full-length percentage of RNA from densitometry plots of gel a, normalized to non-degraded RNA from transcription (see text).

**Fig 7:**
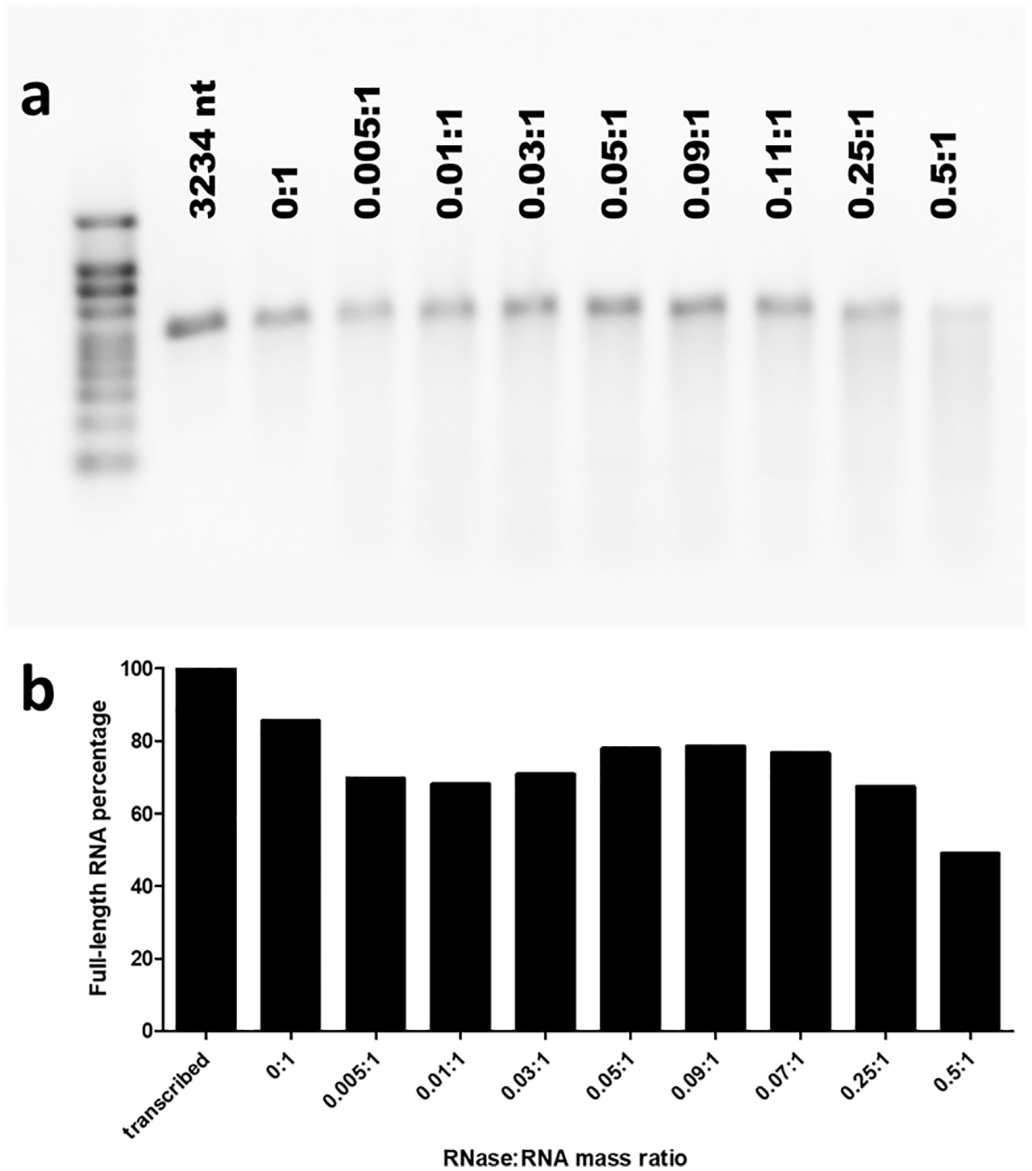
RNA extracted from CCMV VLPs that have been packaged with BMV RNA 1 (3234 nt-long), and RNase-treated at different RNase:RNA mass ratios. a. RNA was extracted from RNase-A treated CCMV VLPs using a Qiagen viral RNA mini kit. 500 ng of each sample were loaded on a 1.2% agarose gel and subjected to electrophoresis for 2 h 15 min at 100 V. The RNA was compared with the NEB^TM^ RNA Markers ssRNA ladder (lane 1). Lane 2 is 500 ng of the in vitro transcribed RNA. Lanes 3-11 show the RNA extracted after treatment with different mass ratios of RNase A. **b.** Full-length percentage of RNA from densitometry plots of gel a, normalized to non- degraded RNA from transcription.

CCMV VLPs containing 4026 nt RNA (Nodamura-RNA1-ferritin linearized with XbaI) were treated for 1 h with different mass ratios of RNase A ranging from 0 to 0.5 μg for every μg of RNA. To determine if the encapsidated RNA was affected by RNase A treatment, agarose gel electrophoresis of the extracted RNA was performed. The results in Fig 6a indicate that a significant percentage of the full-length RNA is protected in CCMV VLPs, with this fraction decreasing for RNase:RNA mass ratios increasing from 0 up through 0.11:1. And at a mass ratio of 0.25:1 and above, essentially no RNA is protected, in contrast with the situation for packaged RNAs that are short enough, i.e., that have lengths less than 3500nt (see Fig 7, below).

Quantification of the extent of protection against RNase, for increasing mass ratios of RNase:RNA was done as follows. From densitometry traces of each lane in the gel, the intensity of the 4026nt band associated with 300 ng of RNA extracted from the RNased sample was compared with that of 300 ng of the purified in vitro transcribed RNA used in the VLP assemblies. The bar plot in Fig 6b shows the intensity of each 4026nt-band associated with RNA extracted from RNase-treated VLPs, normalized to that of unpackaged RNA. The 0:1 RNase:RNA mass ratio sample, for example, in which no RNase is added to the VLPs, shows a 35% loss of intensity relative to unpackaged RNA, because of RNA degradation arising from self-assembly and RNA extraction handling. At the lowest non-zero RNase:RNA mass ratio, 0.005:1, the intensity drops by almost 40%, and then down by another 10–15% as the RNase ratio is increased further.

Fig 7a shows a “titration gel” like that in Fig 6a, except for the packaged RNA being RNA1 of the brome mosaic virus (BMV) with length 3200nt. Here the RNA extracted from VLPs is found to be intact for all RNase:RNA mass ratios up through 0.5: as seen in Fig 7b, for almost all RNase:RNA mass ratios the percentage of full-length RNA that survives assembly and RNase and extraction treatment remains as high as 90% of that for no RNase treatment.

## Conclusions

In this work, RNAs of different lengths (3175, 3234, 3500, 3697, 3799, 3970, 4026, 4197, and 4433 nt) were encapsidated into VLPs using the plant virus CCMV capsid protein. We verified that not all of the RNAs packaged in the CCMV VLPs remain intact after exposure to the endonuclease RNase A. More explicitly, it was demonstrated that RNA up to a length of 3500 nt is fully protected by the CCMV VLP from RNaseA for RNase:RNA mass ratios up through 0.11:1 and that the VLPs containing longer RNA – which include multiplets of capsids – still give significant protection against RNase A. This is an important result since the concentrations of RNases and RNA in the bloodstream [25,27] correspond to mass ratios of this order, implying that the VLPs can protect a good fraction of the full-length RNA, making them functional as RNA delivery systems. Indeed, recent in vivo imaging experiments report [11] strong fluorescence observed in the lymph nodes of mice injected with CCMV VLPs containing ~4000nt- long self-amplifying EYFP mRNA, consistent with the protection afforded by capsid multiplets against physiological RNase levels.

## Supporting information

S1 File Figure S1: RNA transcription. Figure S2: RNA extracted from CCMV VLPs. Figure S3: RNA extracted from RNase A-treated CCMV VLPs. Figure S4. RNA extracted from CCMV VLPs that have been packaged with 4026nt-long RNA, and RNase-treated at different RNase:RNA mass ratios. Figure S5. RNA extracted from CCMV VLPs that have been packaged with BMV RNA 1 (3234 nt-long), and RNase-treated at different RNase:RNA mass ratios.
(DOCX)
