# Supplementary material for: How much genetic information in RNA form can be protected by a CCMV virus-like particle?
Source: PLoS One. 2025 Dec 10;20(12):e0336376. doi: 10.1371/journal.pone.0336376 (PMC12694869; doi:10.1371/journal.pone.0336376)
Supplement: S1 File — Figure S1: RNA transcription. Figure S2: RNA extracted from CCMV VLPs. Figure S3: RNA extracted from RNase A-treated CCMV VLPs. Figure S4. RNA extracted from CCMV VLPs that have been packaged with 4026nt-long RNA, and RNase-treated at different RNase:RNA mass ratios. Figure S5. RNA extracted from CCMV VLPs that have been packaged with BMV RNA 1 (3234 nt-long), and RNase-treated at different RNase:RNA mass ratios. (DOCX) [file pone.0336376.s001.docx]

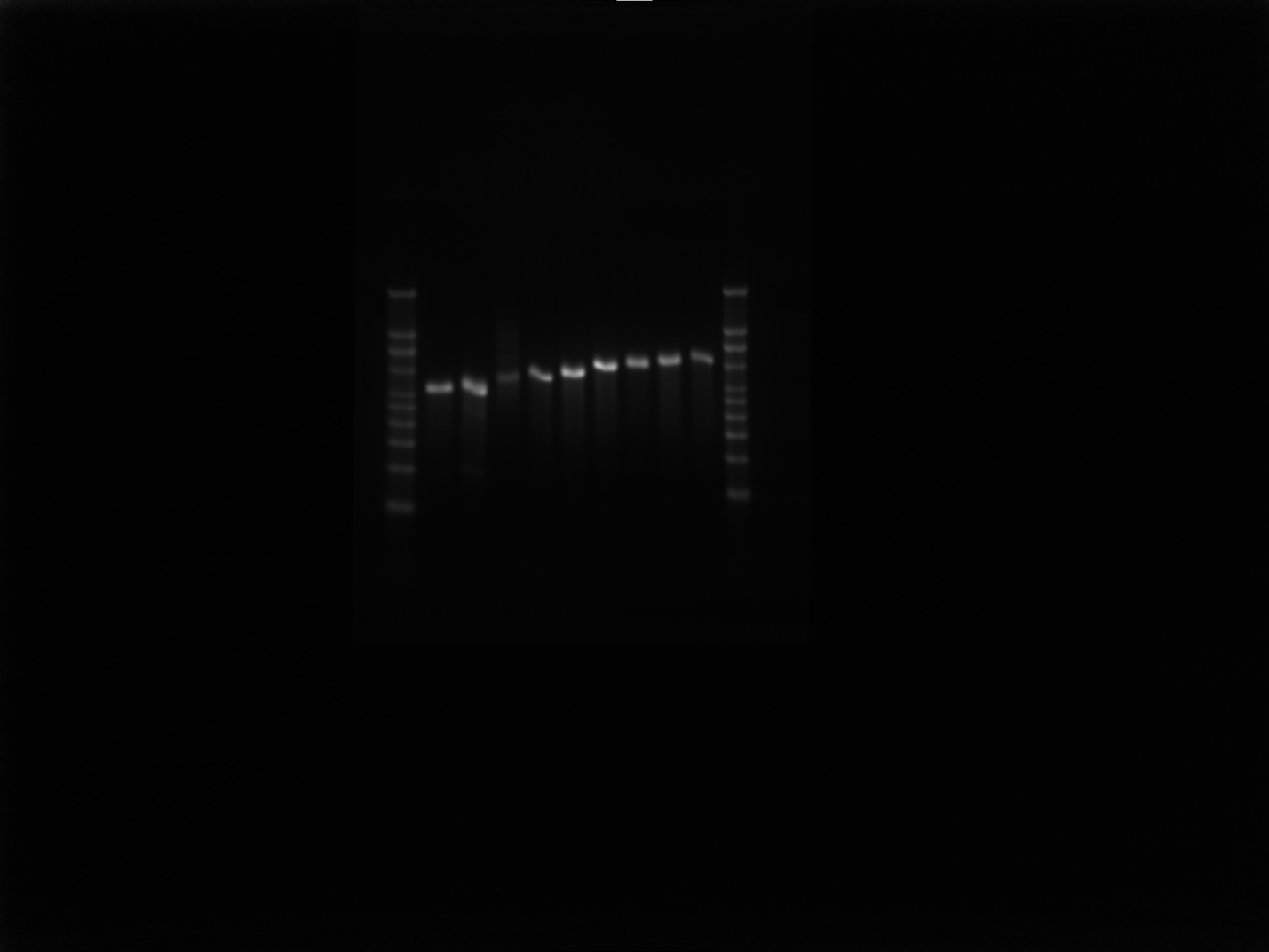


Figure S1: RNA transcription


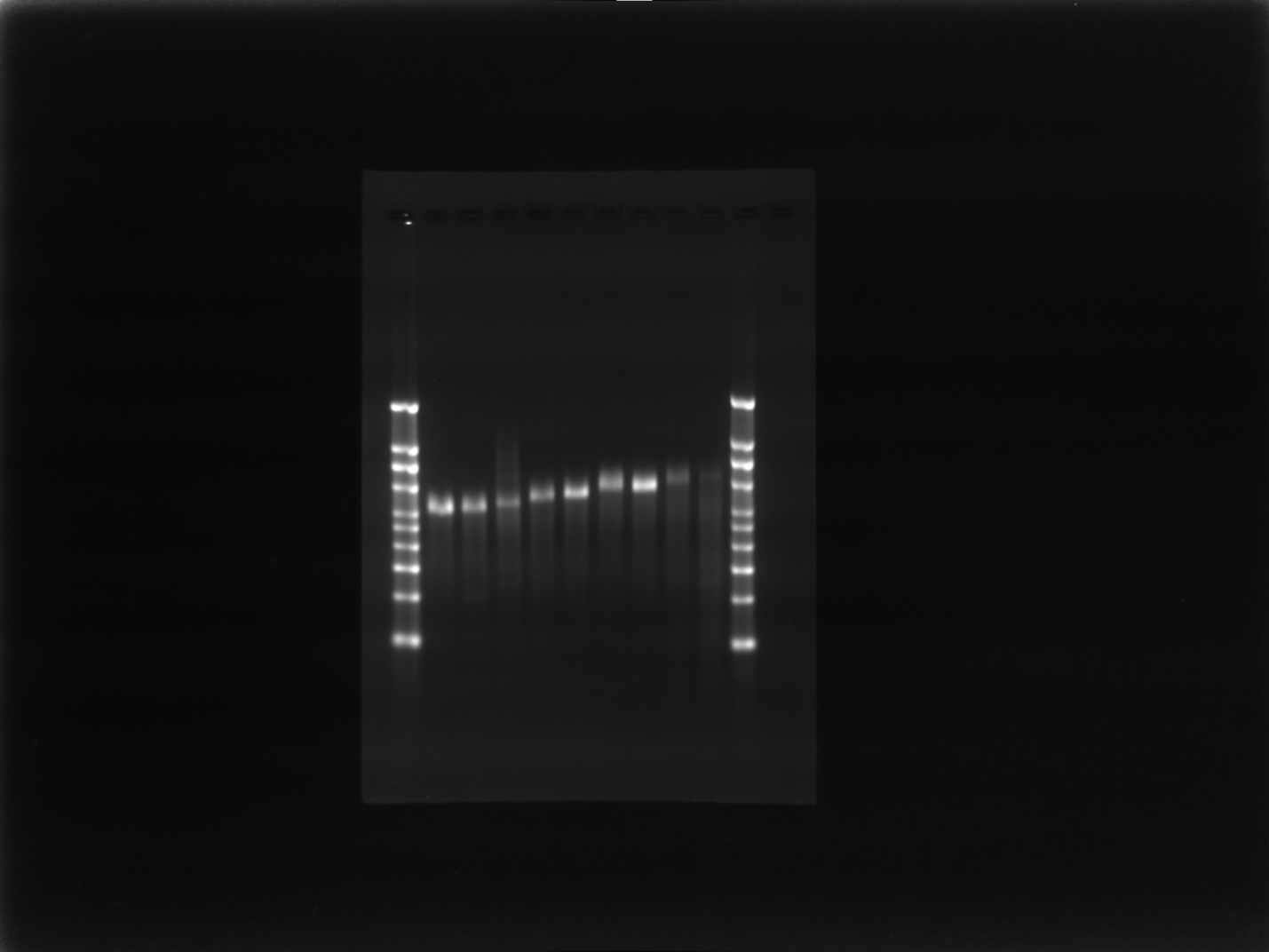


Figure S2: RNA extracted from CCMV VLPs.


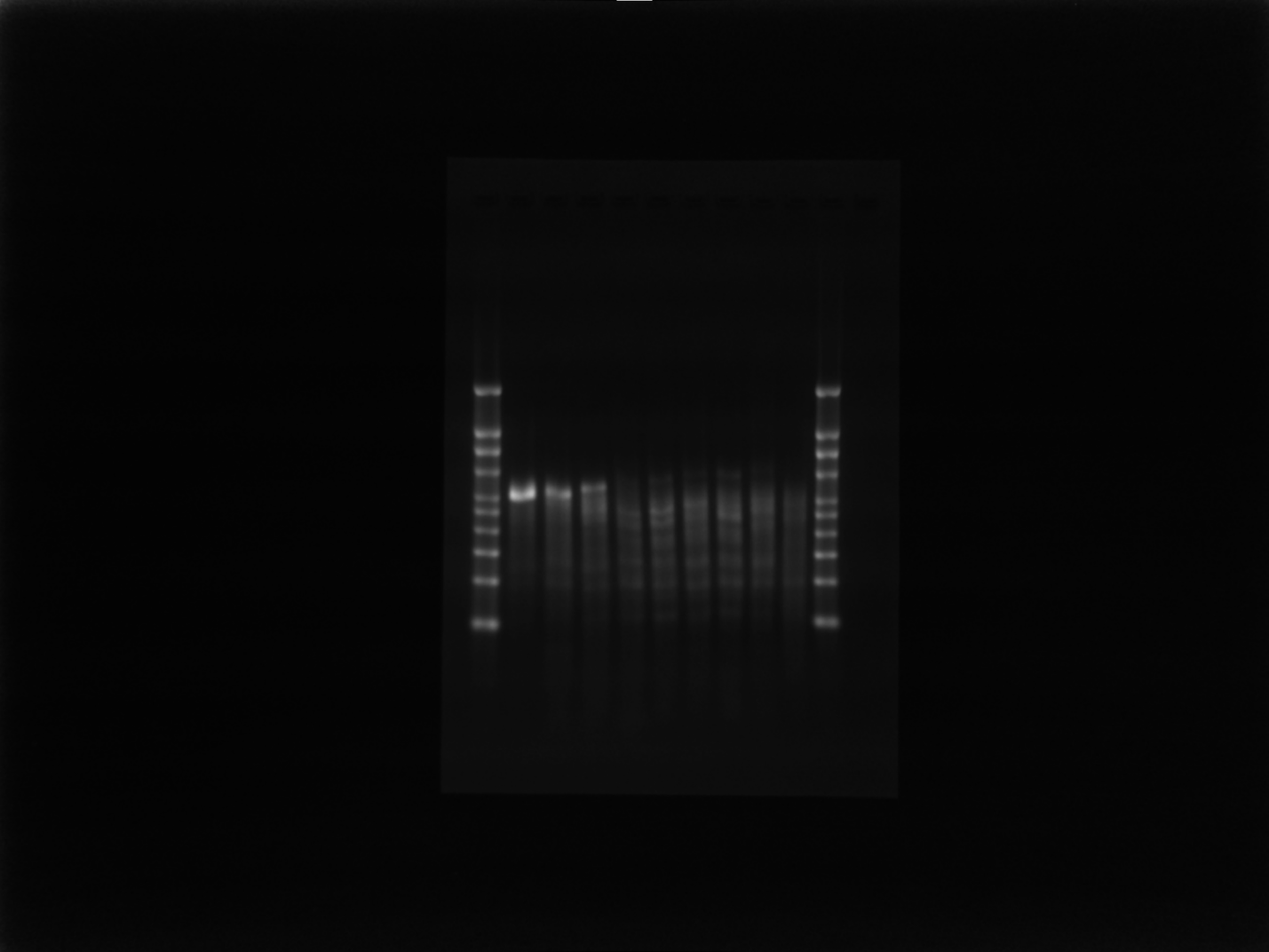


*Figure S3: RNA extracted from RNase A-treated CCMV VLPs.*


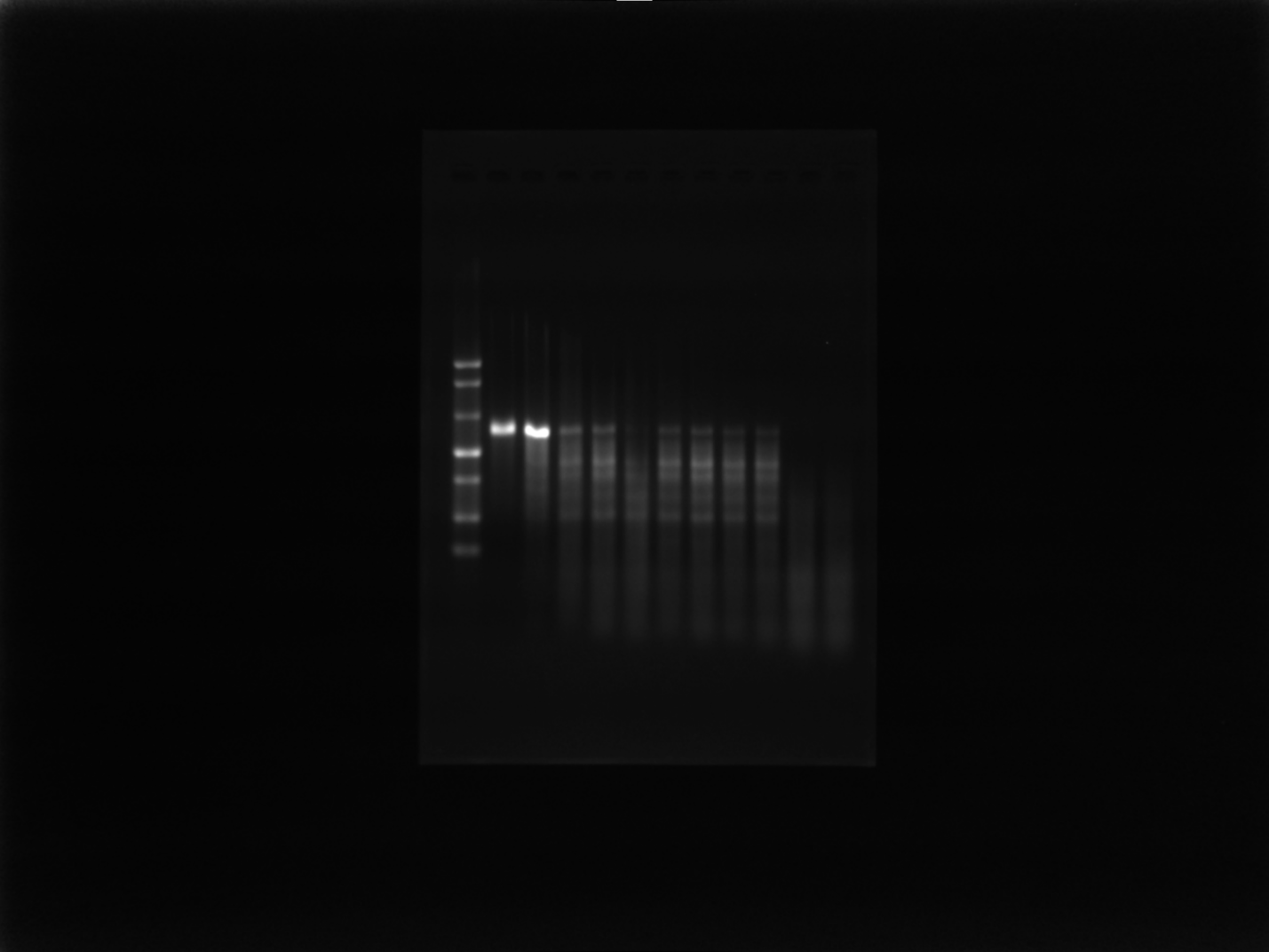


*Figure S4. RNA extracted from CCMV VLPs that have been packaged with 4026nt-long RNA, and RNase-treated at different RNase:RNA mass ratios.*


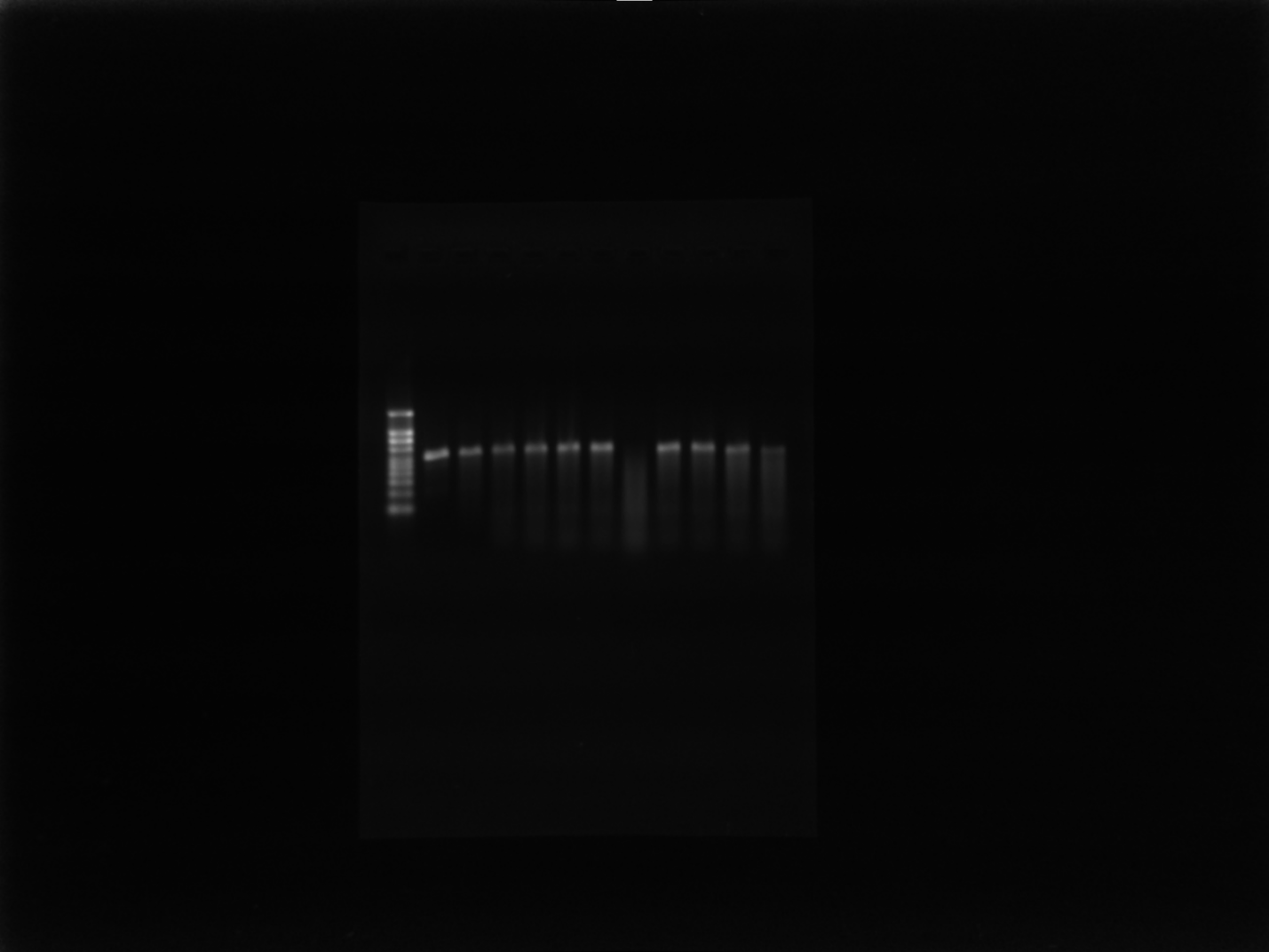


*Figure S5. RNA extracted from CCMV VLPs that have been packaged with BMV RNA 1 (3234 nt-long), and RNase-treated at different RNase:RNA mass ratios.*
